# Road Mortality Contributes to the Evolution of an Urban–Rural Cline in Squirrel Coat Color

**DOI:** 10.1111/eva.70109

**Published:** 2025-05-19

**Authors:** Adam F. Parlin, Bradley J. Cosentino, Richard M. Lehtinen, John E. McDonald, Emma C. C. Sinclair, James P. Gibbs

**Affiliations:** ^1^ Department of Environmental Biology State University of New York College of Environmental Science and Forestry Syracuse New York USA; ^2^ Department of Biology Hobart & William Smith Colleges Geneva New York USA; ^3^ Department of Biology The College of Wooster Wooster Ohio USA; ^4^ Department of Environmental Science Westfield State University Westfield Massachusetts USA; ^5^ Department of Biology Queen's University Kingston Ontario Canada

**Keywords:** adaptive evolution, animal color, citizen science, natural selection, phenotypic variation, road ecology, roadkill, urban evolution, urbanization

## Abstract

Cities impose unique selection pressures on wildlife and generate clines in phenotypic traits along urban–rural gradients. Roads are a widespread feature of human‐dominated landscapes and are known to cause direct wildlife mortality; however, whether they act as a selective force influencing phenotypic trait variation along urban–rural gradients remains unclear. This study tested the hypothesis that roads influence natural selection of coat color in the eastern gray squirrel ( *Sciurus carolinensis* ), a species with two distinct coat colors: a gray morph that is common in all areas and a melanic morph more prevalent in urban areas than in rural ones. Vehicular collisions are a significant cause of mortality in eastern gray squirrels, with the melanic morph more visually conspicuous on roads and more easily detected and avoided by drivers than the gray morph. Standardized road cruise surveys along an urbanization gradient in Syracuse, New York, USA, revealed that the prevalence of melanism among living squirrels in Syracuse was negatively related to distance from the city center, whereas there was no urban–rural cline in melanism among road‐killed individuals, with the melanic morph underrepresented among road‐killed squirrels by up to 30% along the urbanization gradient. An examination of the prevalence of each color morph on and off road surfaces in a range‐wide compilation of > 100,000 photographs of *S. carolinensis* also indicated that the melanic morph was underrepresented among road‐killed squirrels imaged. Our study highlights vehicular collisions as an important source of natural selection on phenotypic traits, suggesting a potential role in shaping patterns of urban evolution and contributing to the maintenance of urban–rural clines.

## 1 Introduction

Roads represent an integral part of connectivity across human‐dominated environments (Coffin 2007) and often have negative ecological effects on wildlife via habitat fragmentation and mortality from vehicular collisions (Forman and Alexander 1998; Seiler 2001; Ramalho and Hobbs 2012; Schwartz et al. 2020). Despite being a major component of environmental change, roads have only recently been recognized as a selection pressure on phenotypic traits (Brady and Richardson 2017). Direct mortality from vehicular collisions can cause natural selection when trait variants affect the probability of collision (e.g., Brown and Brown 2013), and roads can indirectly cause selection by altering habitat along roadways (e.g., contaminants, and vegetation management; Lindberg et al. 2008; Brady and Richardson 2017; Relyea et al. 2024). Road density and traffic volume are known to decrease when moving away from cities (Medley et al. 1995), suggesting that selection from roads could be most prevalent in highly urbanized areas. As such, roads may play an important role in contributing to the development of phenotypic clines along urbanization gradients, which are well documented (Johnson and Munshi‐South 2017; Brady and Richardson 2017).

Our goal was to test whether natural selection from road mortality could contribute to the maintenance of urban–rural clines in phenotypic traits. We used coat color in eastern gray squirrels ( *Sciurus carolinensis* ) as a model system. Gray squirrels have two main color morphs, gray and melanic, caused by a 24‐bp deletion in the melanocortin‐1 receptor gene (*Mc1R* gene; McRobie et al. 2009). The deletion allele is incompletely dominant, such that heterozygotes tend to be brownish‐black, whereas homozygotes for the dominant allele tend to be jet black. In regions where the melanic morph persists, the prevalence of melanism is often negatively related to urban intensity, with urban–rural clines being strongest in large cities with extensive forest cover (Cosentino and Gibbs 2022).

We hypothesize that roads are a novel source of natural selection on squirrel coat color in cities through a process of background matching. Vehicular collisions are a major source of mortality for tree squirrels in cities (Mccleery et al. 2008), and coat color is known to mediate visual detection of *S. carolinensis* on roads. The gray morph is a better match to the color of road pavement than the melanic morph, and human drivers visually detect the melanic morph faster than the gray morph on roads (Gibbs et al. 2019; Bryan 2020; Proctor et al. 2025). Assuming drivers actively avoid hitting squirrels (Crawford and Andrews 2016), greater detectability of the melanic morph may mitigate its risk of collisions with cars more than the gray morph (Gibbs et al. 2019).

We quantified selection on coat color from road mortality by comparing the prevalence of color morphs between road‐killed and living squirrels in Syracuse, New York, USA. Natural selection is thought to maintain a strong urban–rural cline in this city, where survival was greater for the gray than the melanic morph in rural woodlands but similar between morphs in the city (Cosentino et al. 2023). This finding suggests that the selection pressures favoring the gray morph in rural areas are relaxed in the city, or the fitness costs to the melanic morph in rural forests are balanced by novel selection pressures against the gray morph in the city. Here we conduct standardized surveys of roadkill and living squirrels along the urbanization gradient and examined how road and landscape features affect morph‐specific mortality along the survey route. We hypothesized that mortality would be greatest in areas with high traffic and vehicle speeds, the absence of elevated structures for squirrels to cross (e.g., tree branches, power lines), and where forest cover was strongly split across sides of the road (i.e., habitat split; Becker et al. 2007). We also compared the representation of squirrel color morphs between living and road‐killed squirrels across the species' global range using an extensive database of squirrel images derived from a citizen science initiative. Together, these approaches allowed us to evaluate whether vehicular mortality contributes to spatial variation in morph frequencies and acts as a selective agent shaping patterns of urban evolution across both local and global scales.

## 2 Methods

### 2.1 Roadkill Surveys

**FIGURE 1:**
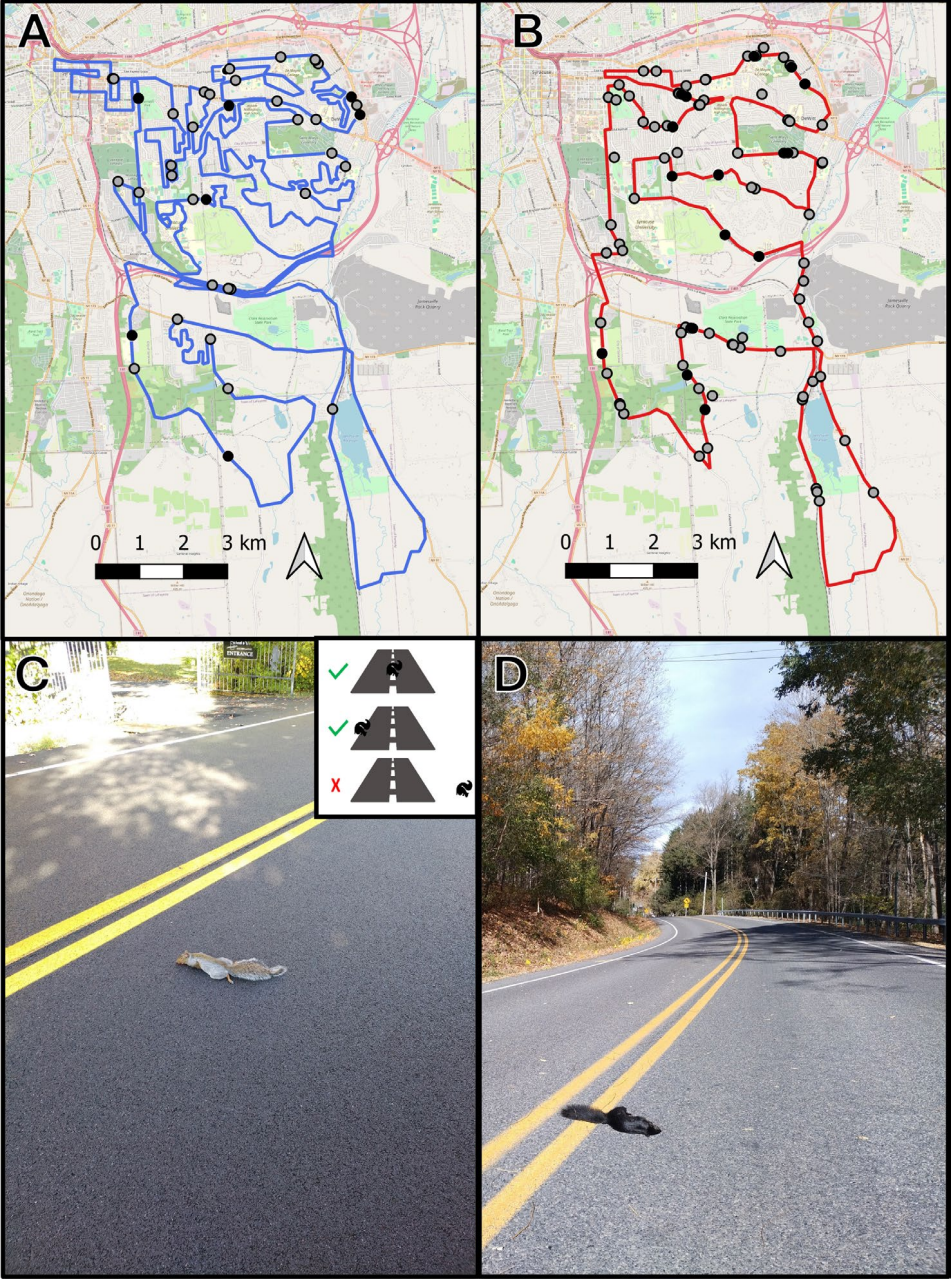
Roadkill survey routes along the urban–rural gradient in Syracuse, NY, USA (43.04, −76.13). (A) Long survey route (130 km) on primary, secondary, and residential roads. (B) Short survey route (65 km) focused more on primary roads. Circles indicate locations of coat‐color specific DOR squirrels (i.e., black = melanic morph, gray = gray morph). (C, D) Example dead‐on‐road (DOR) squirrels recorded during the survey highlighting the contrast of (C) gray and (D) melanic coat colors. Photos by A. Parlin.

We conducted road surveys in Syracuse, New York, USA (43.05, −76.14) in fall (22‐Sep‐2022 to 28‐Oct‐2022) and spring (30‐Mar‐2023 to 17‐May‐2023) when squirrel activity is greatest (Raymond et al. 2021). Two survey routes were established starting near the city center and extending through suburban and residential areas on the peri‐urban fringe (Figure 1A,B). The first route (Figure 1A) was 130 km and surveyed 10 times in fall 2022, whereas the second route (Figure 1B) was 65‐km and surveyed 20 times each in fall 2022 and spring 2023. We had one surveyor for the fall surveys (AFP) and another set of surveyors for the spring surveys who were trained by the fall surveyor. We used a turn‐by‐navigation phone app (Komoot, Potsdam, Germany) to record the survey route and observer speed during each survey. We defined dead‐on‐road (DOR) squirrels as being visible on the paved road (Figure 1C,D). On each survey, we recorded the color morph and location of each carcass with a GPS device (Garmin Etrex; accuracy 3–10‐m). All individuals with brownish‐black to jet black coat were grouped as melanics. Experimental deployments of taxidermy squirrels showed there was no difference in detectability of DOR squirrels by an observer (Text S1). We also estimated how long roadkill persisted along the survey route to ensure only unique DOR locations were recorded—roadkill rarely persisted longer than 1 day (Figure S2). Given the short persistence time of carcasses and that DOR squirrels were geolocated, we suspect there were no duplicate observations of DOR squirrels.

### 2.2 Comparing the Distribution of Color Morphs Between DOR and Living Squirrels

To test for selection on coat color via roadkill, we compared the color morph proportions between DOR squirrels from our driving surveys and living squirrels (e.g., Young et al. 2004; Brown and Brown 2013). Because the prevalence of melanism decreases with increasing distance from the city center (Cosentino et al. 2023), we compared the proportion of melanic individuals between DOR and living squirrels along the urban–rural gradient. We first used logistic regression to estimate the relationship between the proportion of melanic individuals among the DOR squirrels detected and distance from the city center. The color morph *M* of each road‐killed squirrel *i* is a Bernoulli outcome, and we specified a model with the probability of melanism (*p*) among road‐killed individuals as a function of distance to the city center:

$$M_{i}∼\text{Bernoulli}\left(p_{i}\right)$$

$$\text{logit}\left(p_{i}\right)=\alpha +\beta^{*}{\text{distance}}_{i}$$

Next, we estimated the relationship between the proportion of melanic among living squirrels and distance to the city center using a hierarchical model integrating point count and camera trap observations developed by Cosentino et al. (2023). The original model was fit to estimate the urban–rural cline in melanism after accounting for imperfect detection probability using point count and camera trap detections from 76 sites. A single camera trap (Browning Strikeforce Pro XD; Browning Trail Cameras; Utah, USA) was fastened to a tree 30–50 cm off the ground at 49 sites between 2021‐Sep and 2022‐Mar. Cameras were active for 46–379 days (median = 263), with morph‐specific daily detection histories generated by a single observer. Visual point count surveys were conducted at the 49 camera sites and an additional 27 sites without cameras between 2021‐Oct and 2022‐Oct, where the observer recorded the number of melanic and gray squirrels observed in a 3‐min period (2–7 surveys per site, median = 5). We refit the model using data collected by Cosentino et al. (2023) from a subset of 41 sites within a 3‐km buffer of our driving survey routes. Of the 41 sites, 24 sites had camera trap and point count surveys, and 17 sites had only point counts.

The model consists of a submodel to describe the observation process, namely the counts *C*_*ijk*_ and camera trap detections *D*_*ijk*_ based on repeated surveys *j* at each location *i* for each color morph *k*:

$$C_{\mathrm{ijk}}∼\text{Binomial}\left(N_{\mathrm{ik},}\;p_{\mathrm{ijk}}\right)$$

$$D_{\mathrm{ijk}}∼\text{Bernoulli}\left({p^{*}}_{\mathrm{ijk}}\right)$$

where *N*_*ik*_ are the latent morph abundances at each site, *p*_*ijk*_ is the individual detection probability, and p*_ijk_ is the probability of detecting at least one individual of each morph at a site during each survey: p*_ijk_ = 1 − (1 − *p*_*ijk*_)^*Nik*^. Following Cosentino et al. (2023), we modeled *p*_*ijk*_ as a quadratic function of mean daily temperature using temperature data from a nearby weather station (Syracuse Hancock International Airport; Network: GHCND; ID: USW00014771):

$$\text{logit}\left(p_{\mathrm{ijk}}\right)=a0_{k}+a{1_{k}}^{*}{\text{temperature}}_{\mathrm{ij}}+a{2_{k}}^{*}{{\text{temperature}}^{2}}_{\mathrm{ij}}$$

Two additional submodels were used to describe total abundance of squirrels (N_total_*i*_) and proportion of melanic squirrels (*p*_melanic_*i*_) as a function of distance to city center:

$$N\_{\text{total}}_{i}\sim \text{Poisson}\left(\lambda_{i}\right)$$

$$\log\left(\lambda_{i}\right)=\text{β0}+\beta 1^{*}{\text{distance}}_{i}$$

$$N_{\text{i1}}\sim \text{Binomial}\left(N\_{\text{total}}_{i},p\_{\text{melanic}}_{i}\right)$$

$$\text{logit}\left(p\_\text{melani}c_{i}\right)=\text{β0}+\beta 1^{*}{\text{distance}}_{i}$$

where λ_*i*_ is the mean expected total abundance of squirrels at each site.

We used the *jagsUI* R package (Kellner and Meredith 2021) and JAGS 4.1.0 (Plummer 2017) in R (Version 4.2.0; R Core Team 2022) to fit the models with Bayesian inference and Markov chain Monte Carlo. Distance to city center and temperature covariates were standardized (mean = 0, SD = 1) before fitting the model. We used noninformative normal prior distributions for regression coefficients, with the exception of uniform distributions constraining mean detection probability, mean total squirrel abundance, and mean proportion melanic to realistic values based on prior knowledge (see code in https://doi.org/10.5281/zenodo.15249929 for specific priors for each parameter). Each model was fit with three chains with 20,000 adaptation iterations followed by 12,000 iterations, discarding the first 1000 iterations as burn‐in and thinning the remaining iterations by 10. The resulting 3000 iterations were used to describe the posterior distribution for each parameter. We confirmed convergence of parameter estimates with the Gelman–Rubin statistic (R‐hat < 1.1; Gelman and Hill 2007).

We used the models to derive estimates of proportion melanic for living and DOR squirrels along the range of distances from city center (0.93–11.3‐km). We then quantified the difference in proportion melanic between living and DOR squirrels at each distance. This process was repeated for each iteration to generate a posterior distribution for the difference in proportion melanic between living and DOR squirrels at each distance. We summarized the posterior distribution by quantifying the mean and a 95% credible interval for the difference in proportion melanic at each distance.

### 2.3 Landscape Predictors of Mortality Risk

We tested whether road mortality risk for each morph was related to road and landscape features of interest, including speed limit, traffic volume, presence of crossing structures (e.g., utility wires), and the degree of habitat split (Becker et al. 2007) across roads. We first developed a directed acyclic graph (DAG) showing hypothesized causal relationships of these variables to components of landscape change in the broader urban environment. The goal of using the DAG was to identify potential confounds that could generate spurious associations or mask relationships between road mortality risk and the predictors of interest (McElreath 2020). We created a DAG for each color morph (Figure S1), each based on a structural equation model developed by Borden et al. (2024) to understand drivers of spatial variation in abundance of each color morph in our study area.

The foundation of each DAG consisted of hypothesized causal pathways among distance to city center, human population density, building density, forest cover, forest fragmentation, and the local abundance of each color morph (Figure S1). These relationships are empirically supported in our study system (Borden et al. 2024). We then added hypothesized causal relationships between these variables and the road and landscape features predicted to affect road mortality risk. We expected human population density to affect speed limits and traffic volume, and we included a direct effect of building density on the number of above‐ground crossings (e.g., utility lines). Because overhanging branches can provide above‐ground road crossings, we also included a direct effect of forest cover on crossings. The degree of habitat split, which we defined as the difference in forest cover across roads, was expected to be more common in areas of high forest fragmentation, so we included a direct effect of fragmentation on habitat split. Finally, we included direct effects of the abundance of each color morph on road mortality risk, as numerically more road mortalities are expected where squirrels are more abundant.

For each DOR location (*n* = 141), we generated individual buffers (300‐m) representing the typical home‐range size of gray squirrels (< 0.5 ha; Koprowski et al. 2016). For comparison, we also generated a matching number of reference locations and buffers where DOR squirrels were not observed during our surveys. The reference locations were sampled randomly using the *st_sample()* function from the *sf* R package (Pebesma 2018). We then quantified road and landscape features for each DOR and reference location.

We quantified habitat split as the difference in forest cover between each half of the 300‐m buffer on opposite sides of the road. We extracted the tree cover layer from the European Space Agency's (ESA) WorldCover dataset (10‐m resolution; Venter et al. 2022) to estimate forest cover and habitat split. For each DOR and reference site, we determined the presence or absence of potential road crossing structures by identifying either powerlines or tree canopy crossing the road from a Google panorama view. Speed limit and annual average daily traffic (AADT) were recorded from a New York State Department of Transportation (NYSDOT) dataset (https://catalog.data.gov/dataset/nys‐traffic‐data‐viewer). When the posted speed limit was not available in the NYSDOT dataset, we estimated the driving speed (miles per hour) at each DOR and reference location based on the Komoot app, which logged the GPS location and time during each survey permitting calculation of speed along the route. For segments of roads without AADT defined by the NYSDOT data, we used kriging based on MAF/TIGER Feature Class Code (MFTCC) road type using the *gstat* R package (Pebesma 2004; Gräler et al. 2016). We used a variogram analysis on the logarithmically transformed AADT values to assess the spatial correlation structure. We then fit the sample variogram using a spherical model with a nugget parameter of 0.75 and a range parameter of 1. The model was then used to interpolate missing AADT values over a regular grid covering the study area. Finally, the kriged AADT estimates were rasterized onto a raster grid and extracted for missing AADT values.

We used logistic regression to estimate the relationship of road mortality risk for each color morph to each explicit predictor of interest (Rohrer 2018). For each direct effect of interest, we used the *dagitty* R package (Textor et al. 2016) to identify the appropriate adjustment set based on the DAG. For example, to test for the direct effect of speed limit on mortality risk, we adjusted for human population density, which is a confounder (Figure S1). Human population density, total forest cover, and forest fragmentation were required as adjustment covariates. Human population density was quantified for each 300‐m buffer using NASA's CIESIN gridded human population dataset (Center for International Earth Science Information Network (CIESIN), Columbia University 2018). Forest cover was quantified as the proportion forest in the entire 300‐m buffer. For forest fragmentation, we used the *landscapemetrics* R package (Hesselbarth et al. 2019) to quantify the number of disjunct core areas in a 1‐km buffer around each DOR and reference location. We used the *brglm2* R package (Kosmidis 2023) to fit the logistic regression models with the *brglm_fit()* function using Firth's method for mean bias reduction. Human population density, forest fragmentation, and AADT were all log‐transformed to meet the assumption of log‐odds linearity.

### 2.4 Squirrel Mortality Across Population Range

To examine whether road mortality risk was contingent on color morph throughout the global range of the eastern gray squirrel, we leveraged images of squirrels taken by citizen scientists from *iNaturalist* via the *SquirrelMapper* citizen science program (https://www.squirrelmapper.org). All images submitted through 13‐Nov‐2022 and classified as research grade were included for analysis (*N* = 126,193). For each image from *iNaturalist*, we used the *Zooniverse* platform to crowdsource the classification of the number of squirrels (zero, one, or two or more), coat color (melanic, gray, other, or unclear), status (living or dead), and surface on which the squirrel was photographed (road or other). Images were classified by a minimum of 10 participants, and we retained images with a minimum agreement threshold of 80% for number of squirrels, coat color, status, and surface (Cosentino and Gibbs 2022). We restricted analyses to photos classified as having a single squirrel that was either gray or melanic (Melanic: *N* = 12,705; Gray: *N* = 96,025). Although the *SquirrelMapper* observations have a global distribution and span both urban and rural habitats, it is important to note that most observations are from urban areas where human population density (i.e., observers) is highest.

We compared the proportion of squirrels photographed dead between color morphs in two ways. First, we compared the proportion of DOR squirrels relative to all living squirrels for each color morph (*N* = 108,730 squirrel observations). Second, we subset all squirrels photographed on a road to compare the proportion of DOR squirrels between morphs relative to squirrels photographed alive on roads (*N* = 1473 squirrel observations). We compared the probability of a squirrel being photographed dead on a road between color morphs for each comparison using a Pearson's *χ* ^2^ test with Yates' continuity correction with the *stats* R package (R Core Team 2022). We then used a Cramer's V from the *rcompanion* R package (Mangiafico 2022) to determine effect size for each *χ* ^2^ test.

## 3 Results

**FIGURE 2:**
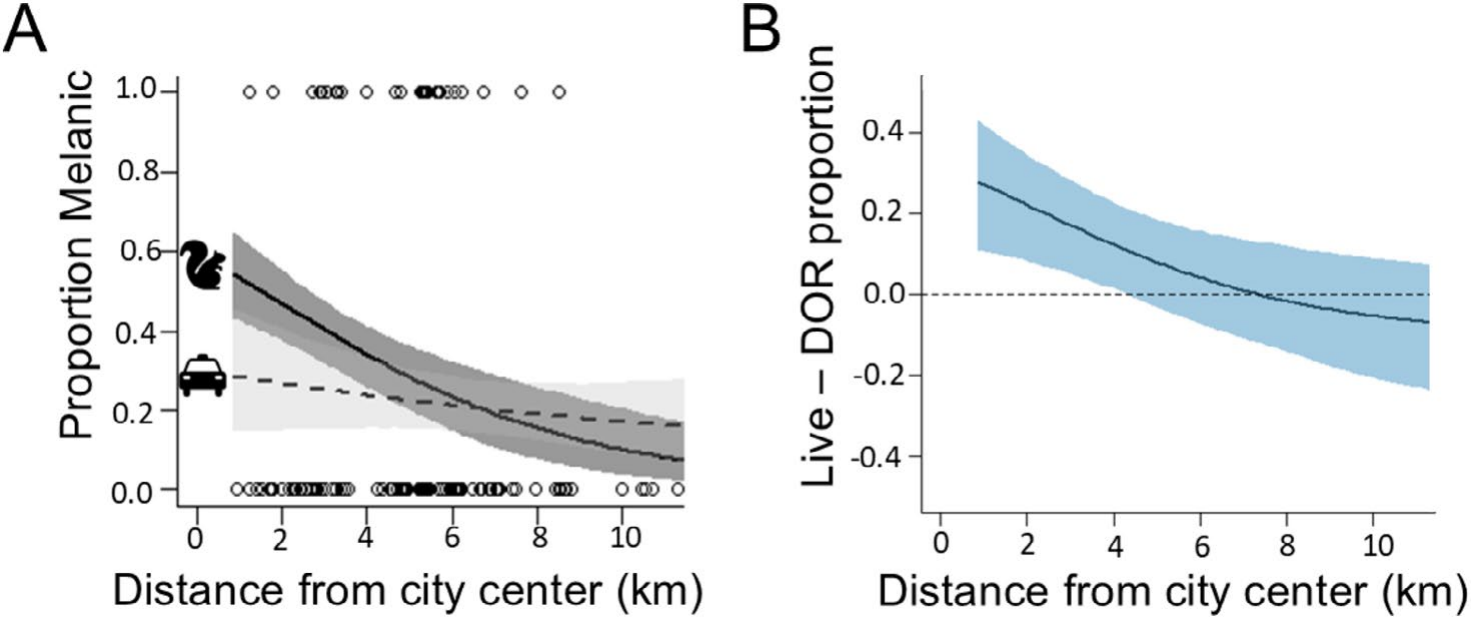
Dead‐on‐road squirrels along urban–rural gradient for all squirrels (1 = melanic, 0 = gray). (A) Relationship of proportion melanic of eastern gray squirrels ( *Sciurus carolinensis* ) to distance from city center for living (dark gray) and DOR (light gray) squirrels, and (B) difference in the estimated proportion melanic between living and DOR squirrels along the urbanization gradient (B). Solid lines are posterior means and shaded areas are 95% credible intervals.

Across our fall and spring road surveys, we encountered 112 gray and 29 melanic squirrels dead on the road surface. The proportion of squirrels that were melanic declined with increasing distance from city center for living squirrels but not DOR squirrels (Table 1; Figure 2A). The proportion of melanic DOR individuals was underrepresented by nearly 30% relative to the living proportion of melanic at the city center, and underrepresentation of melanics was detectable up to ~4.5 km from the city center (Figure 2B).

**TABLE 1:** Parameter estimates and 95% Bayesian credible intervals (CI) for models of total abundance, proportion melanic, and detection probability of eastern gray squirrels ( *Sciurus carolinensis* ).

Dataset	Response variable	Parameter	Mean	95% LCI	95% UCI
Living squirrels	Total abundance	Intercept	2.58	2.40	2.80
		Distance to city center	**−0.62**	**−0.78**	**−0.47**
	Proportion melanic	Intercept	**−0.85**	**−1.28**	**−0.48**
		Distance to city center	**−0.80**	**−1.22**	**−0.40**
	Melanic detection probability	Intercept	**−2.16**	**−2.32**	**−1.97**
		Temperature	**−0.058**	**−0.11**	**−0.0023**
		Temperature^2^	**−0.34**	**−0.39**	**−0.28**
	Gray detection probability	Intercept	**−1.53**	**−1.84**	**−1.39**
		Temperature	−0.019	−0.060	0.021
		Temperature^2^	**−0.39**	**−0.43**	**−0.35**
Dead on road squirrels	Proportion melanic	Intercept	**−1.36**	**−1.79**	**−1.36**
		Distance to city center	−0.22	−0.67	0.1

*Note:* The relationship of proportion melanic to distance of city center was estimated with separate models for living squirrels and DOR squirrel observations. For living squirrels, a hierarchical model was used that accounted for detection probability of each morph during surveys. Credible intervals that do not overlap 0 are indicated in bold.

**FIGURE 3:**
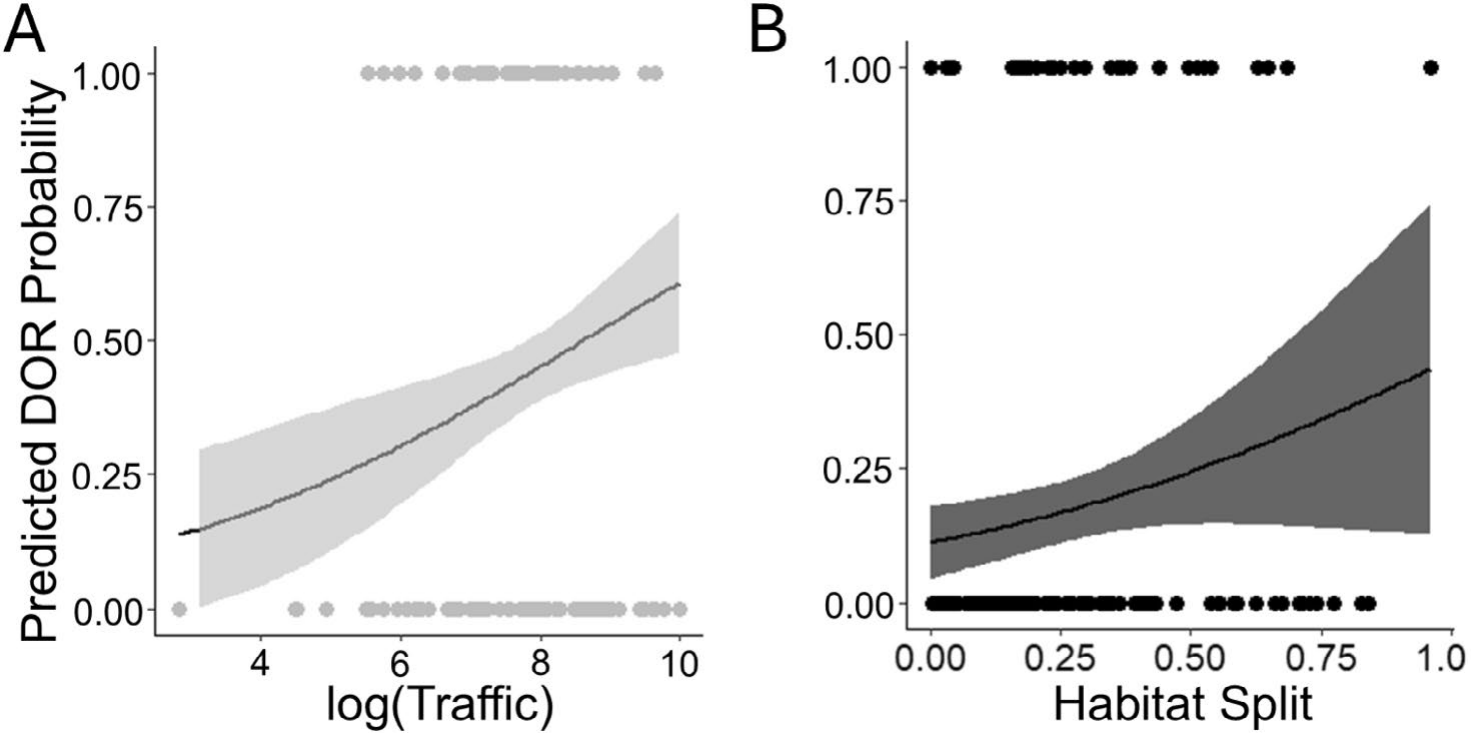
Relationship of probability of roadkill mortality to (A) traffic volume for the gray morph and (B) habitat split for the melanic morph. Points represent observed mortalities (1) and reference locations without mortalities (0). For the gray morph, the traffic (log‐transformed) as a covariate of interest had a significant effect (β = 0.32 ± 0.12, *p* < 0.01), while for the melanic morph it was the habitat split as the covariate of interest having a significant effect (β = 1.89 ± 0.92, *p* < 0.05). Predicted probability of roadkill mortality (solid line) and 95% confidence interval (shading) are from logistic regression models (Table S1).

Our models revealed moderate differences between morphs in how the road and surrounding landscape characteristics affect road mortality risk. Mortality risk for the gray morph was positively related to traffic volume (Figure 3A; Table S1) and presence of aboveground road crossings (Table S1), whereas for the melanic morph the probability of mortality was positively but weakly related to the degree of habitat split (Figure 3B; Table S1). The remaining variables did not predict DOR probability when conditioning on appropriate adjustment covariates (Table S1).

**FIGURE 4:**
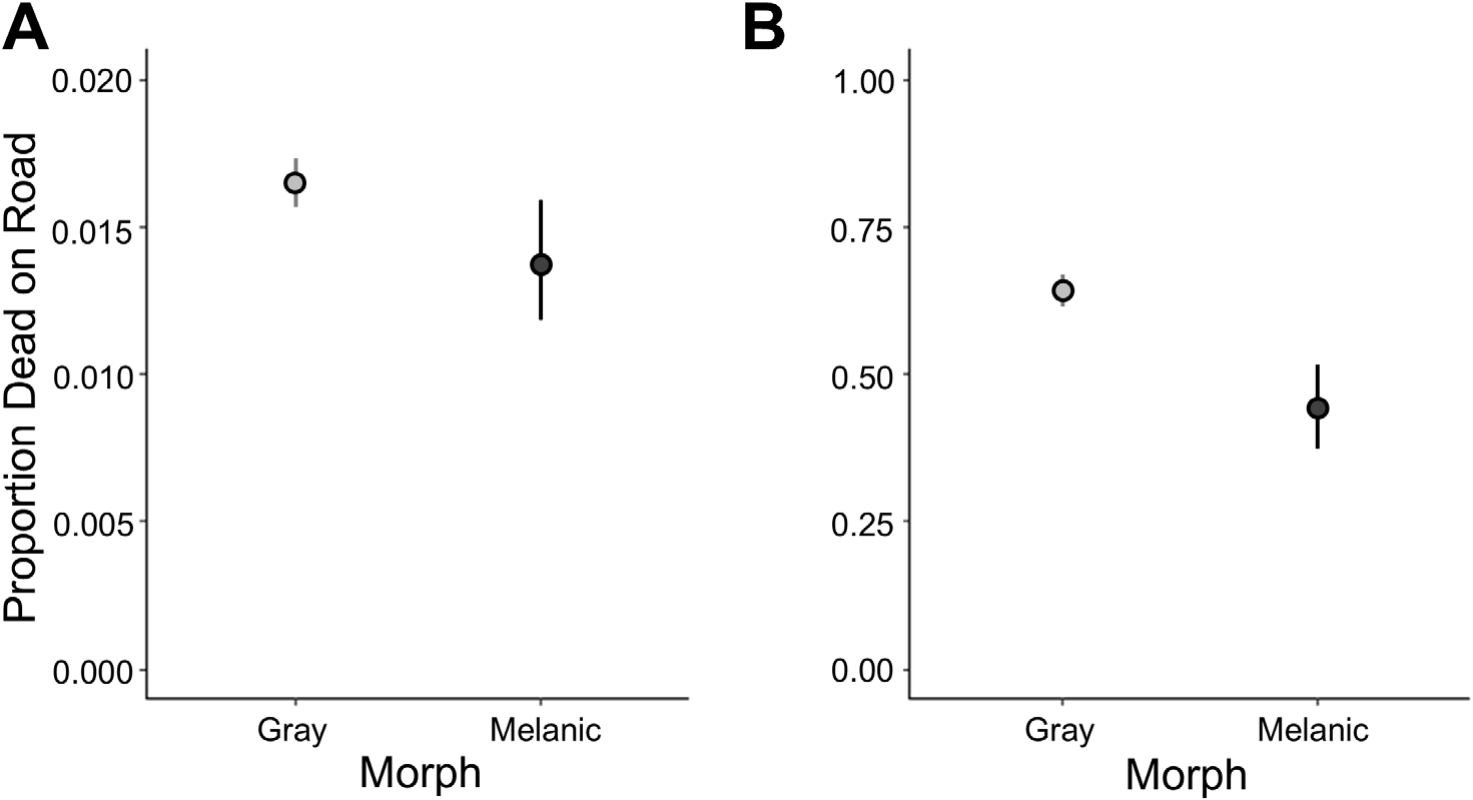
Proportion squirrels photographed dead on a road (DOR) by color morph (gray, melanic) among (A) all observations of eastern gray squirrels ( *Sciurus carolinensis* ) in the iNaturalist dataset (*N* = 108,730) regardless of surface type and (B) only observations of squirrels on roads (*N* = 1473). (A) For all observations of gray squirrels DOR melanic squirrels were underrepresented, while DOR gray squirrels were overrepresented ($\chi_{1}^{2}$ = 5.24, *p* = 0.02, Cramer's V = 0.01). (B) For the subset of squirrels photographed on roads, melanic were more underrepresented ($\chi_{1}^{2}$ = 25.78, *p* < 0.001, Cramer's V: 0.13). Error bars represent 95% confidence intervals estimated with the Agresti‐Coull method.

In the citizen science dataset of photographed squirrels, DOR melanic squirrels were underrepresented (observed = 174.0, expected = 205.7) and DOR gray squirrels were overrepresented (observed = 1581.0, expected = 1549.9) relative to squirrels photographed alive ($\chi_{1}^{2}$ = 5.24, *p* = 0.02; Figure 4A), albeit weakly (Cramer's V: 0.01). For the subset of squirrels photographed on roads, melanic squirrels were also underrepresented ($\chi_{1}^{2}$ = 25.78, *p* < 0.001, Cramer's V: 0.13; Figure 4B).

## 4 Discussion

We found the prevalence of melanism in *S. carolinensis* declined moving away from Syracuse, New York, USA for living squirrels, as expected given the strong urban–rural cline in melanism (Cosentino et al. 2023). However, there was no urban–rural cline in melanism among road‐killed squirrels when compared against the proportion of living gray and melanic coat morphs from our camera trap network. Our study shows the melanic morph was underrepresented among roadkill relative to living morph proportions in densely urbanized areas, where the strength of selection from roads is likely strongest due to increased traffic and road density. Moreover, we found a signal of the melanic morph being underrepresented among DOR squirrels in a global dataset of *S. carolinensis* photographs taken by citizen scientists. Collectively, our results support the hypothesis that roads can be an important cause of natural selection on phenotypic trait variation and likely contribute to the maintenance of urban–rural clines in the melanism of gray squirrels.

Our findings indicate that the role of natural selection in maintaining urban–rural clines is complex. Previous work on the urban–rural cline in melanism in Syracuse highlighted the difference in survival between the melanic and gray morphs in rural areas, where selection acts against the melanic morph (Cosentino et al. 2023). In contrast, there was no difference in survival between color morphs in the urban environment, leading to the hypothesis that the selection pressures against the melanic morph in rural areas are relaxed in the city. Our study shows this is not necessarily the case. It is possible the selection pressures against the melanic morph in rural landscapes persist in urban areas but are balanced by the road‐induced selection against the gray morph that we document here—thus leading to no net selection on coat color in the city. The underrepresentation of the melanic morph among roadkill persists only at the urban end of the gradient, suggesting that the urban–rural cline in melanism may be explained in part by the relaxation of selection from roads moving *away* from the city. Vehicular collisions are thought to be the primary cause of tree squirrel mortality in cities, whereas predation is often the primary driver of mortality in rural areas (e.g., Mccleery et al. 2008). For humans, the melanic morph is more visible than the gray morph not only on roads, but also in forested and urban greenspace habitats (Proctor et al. 2025). One possibility is that selection from predation acts against the less cryptic melanic morph across the urbanization gradient, but is counterbalanced in cities by a combination of relatively weaker predator‐induced selection and road‐induced selection against the gray morph.

What explains the underrepresentation of the melanic morph among roadkill? Human drivers generally try to avoid colliding with wildlife on roads, particularly mammals (Beckmann and Shine 2011; Crawford and Andrews 2016). Given that melanic squirrels are more visible than their gray counterpart on asphalt (Proctor et al. 2025), one potential hypothesis is that earlier detection gives human drivers more reaction time to a squirrel on the road. The analysis of *iNaturalist* observations supports this interpretation: melanic road‐killed squirrels were more strongly underrepresented among the pool of squirrels observed on roads (i.e., only those squirrels that attempted to cross a road) than among all squirrel observations regardless of location. However, it is also possible that behavioral differences between squirrel color morphs contribute to differential probability of vehicular collisions, such as a tendency to continue across the road, run parallel with the car, or turn back (AFP; Pers. Obs.). Wildlife species vary in their behavioral response to linear landscape features such as roads (e.g., Gibbs 1998; Hibbitts et al. 2017; Dickie et al. 2020) but less is known about intraspecific variation in road crossing behavior. Melanism in animals has been found to influence stress response, whereby darker individuals are less sensitive to stressful factors (Ducrest et al. 2008). Another explanation could be differential diurnal activity patterns between the color morphs, linked to homeostatic and energy maintenance (Ducrest et al. 2008). The detection submodel for living squirrels showed that individual detection probability was lower for the melanic than the gray morph (Table 1). The camera trap dataset could be leveraged to test whether the melanic morph in urban environments has a more confined activity period, which could contribute to the melanic morph spending less time on roads than the gray morph, or is active during times of day with lower traffic volume. Fine‐scale tracking or movement experiments would also be insightful to test whether the melanic morph is either less likely to cross roads, or more likely to use overhead crossing structures than the gray morph.

Our analyses provided some insight into spatial variation in road mortality risk for each color morph. The probability of road mortality for the gray morph tended to be greatest at sites with high traffic volume and the presence of overhead crossing structures, whereas road mortality risk for the melanic morph was positively related to habitat split. In locations with higher traffic volume, it is likely that the probability of mortality increases with every road crossing (Bencin et al. 2019). The relationship of roadkill risk to traffic volume for the gray but not melanic morph suggests the melanic morph may indeed be less likely to cross roads than the gray morph, but this remains to be explicitly tested. The relatively greater risk of roadkill for the gray morph at sites with overhead structures may also point to a low propensity for using overhead structures by the gray morph. Gap traversability in squirrels is largely influenced by gap distance and the compliance of the leaping structure (gray morph—Hunt et al. 2021). Although above‐ground crossings should reduce road mortality risk, it is possible that certain crossing structures, such as telephone lines, may be unstable while squirrels are crossing and are commonly avoided. The positive relationship of melanic road mortality to habitat split could be due to differences in resource provisioning on either side of the road where the squirrel was hit, whether the resources are anthropogenic or natural food sources (Hill et al. 2021). However, whether melanic squirrels are more likely to provision on either side of a habitat split than their gray counterparts, or gray squirrels are less likely to use above‐ground crossing structures to cross roads, remains to be explored. It is worth noting the relationships of gray mortality to overhead structures and melanic mortality to habitat split were relatively weak. Detailed studies of fine‐scale movement and monitoring will provide more insight into the behavioral differences between melanic and gray squirrels around roads.

This study sheds light on the complex interplay between urbanization and novel adaptive drivers of phenotypic trait variation. Transportation infrastructure, notably roads, presents a significant challenge to wildlife, with vehicular collisions being a major source of mortality (Schwartz et al. 2020). Our results demonstrate how direct mortality from roads can translate into a significant source of natural selection. The underrepresentation of melanic squirrels based on standardized surveys in Syracuse, but also at a global scale from the citizen science dataset, reinforces the findings and potential implications for urban evolution in cities with dense road networks. The ubiquity of roads and likely tendency for road‐induced mortality to be strongest at the urban extreme suggests roads may play a general role in contributing to the maintenance of urban–rural clines in phenotypic traits of wildlife through natural selection. Our work highlights the importance of linking trait variation to fitness in the context of multiple ecological selection pressures to understand the role of selection in maintaining urban–rural clines (Johnson and Munshi‐South 2017).

## Conflicts of Interest

The authors declare no conflicts of interest.

## Supporting information


Appendix S1.

## Data Availability

Data and corresponding R code is uploaded to the following GitHub: https://github.com/bcosentino/squirrel‐road‐selection.git. Data and code have also been deposited to https://doi.org/10.5281/zenodo.15249929.
