# Supplementary material for: Road Mortality Contributes to the Evolution of an Urban–Rural Cline in Squirrel Coat Color
Source: Evol Appl. 2025 May 19;18(5):e70109. doi: 10.1111/eva.70109 (PMC12089062; doi:10.1111/eva.70109)
Supplement: Supplementary file 1 — Appendix S1. [file EVA-18-e70109-s001.docx]

**SUPPLEMENTARY MATERIALS**

**Text S1.** Our aim during the road cruise surveys beyond the counting of morph-specific dead-on-road (DOR) was to (1) assess the surveyor’s ability to detect DOR gray and melanic morph squirrels and (2) determine how long carcasses persisted along the survey route to ensure only unique DOR locations were recorded.

*Squirrel roadkill morph detectability. –* To assess the surveyor’s ability to detect carcasses of gray or melanic morph, we conducted standardized surveys using taxidermy models. A single individual randomly dropped taxidermy squirrel models (gray = 10; melanic = 10) along a 10km route in areas similar to observed carcasses. The surveyor then drove along the route and picked up the taxidermy squirrels. After each run, we counted the number of missing morph-specific squirrels. These routes were conducted three times under similar weather conditions as the road surveys. The detectability of taxidermy models from the three trial runs yielded a 96.6% detection for melanic (29 out of 30) and 90% detection for gray (25 out of 30).

*Roadkill Longevity. –* Prior to each survey, we made note of the previous locations where dead squirrels were located to determine carcass persistence under natural conditions. We then determined the persistence of roadkill for squirrels in general, and morph-specific persistence, using a Kaplan-Meier curve from the *survival* R package (Therneau and Lumley, 2015). To assess the differences in morph-specific carcass persistence, we compared the longevity of melanic and gray morphs using a log-rank test for individuals that persisted more than a day. We focused on the fall road cruise surveys as these were done more consecutively than during the spring, broader road cruise surveys. We found that squirrel carcasses, whether the gray or melanic morph, rarely persisted longer than a day (Suppl. Fig. 2A), with only a few instances of multi-day persistence along high traffic areas such as a highway (Pers. Obs.). We also found no difference between the persistence of specific DOR color morphs (Suppl. Fig. 2B; Cox proportional hazard: *X*^2^_1_ = 0.82, P > 0.05).

**SUPPLEMENTAL TEXT REFERENCES**

Therneau, T. M. & Lumley, T. (2015). Package ‘survival’. *R Top Doc*, **128**, 28-33.

**SUPPLEMENTAL TABLE**

**Table S1.** Model output for logistic regressions for predictors of spatial variation in roadkill for each color morph. Bold in the model indicates the covariate of interest, whereas italics represents covariates that were included as adjustment covariates based on the directed acyclic graph (Suppl. Fig. 1). Bold P-values indicate a significant effect of the variable of interest.

| **Morph** | **Model** | **Parameter of Interest** | **Other Parameters** | **Estimate** | **Standard Error** | **P-value** |
| --- | --- | --- | --- | --- | --- | --- |
| Gray |  |  | (Intercept) | -2.5 | 1.08 | 0.021 |
|  | Gray mortality ~ **Speed** + Human Density | *Speed* | *-* | *0.01* | *0.01* | *0.388* |
|  |  |  | Human Density | 0.29 | 0.11 | 0.008 |
|  |  |  | (Intercept) | -4.0 | 1.07 | < 0.001 |
|  | Gray mortality ~ **Traffic** + Human Density | *Traffic* | *-* | *0.32* | *0.12* | ***0.008*** |
|  |  |  | Human Density | 0.2 | 0.09 | 0.027 |
|  |  |  | (Intercept) | -5.02 | 0.94 | < 0.001 |
|  | Gray Mortality ~ **Crossings** + Human Density + Forest Cover | *Crossing* | *-* | *0.66* | *0.33* | ***0.042*** |
|  |  |  | Human Density | 0.38 | 0.11 | < 0.001 |
|  |  |  | Forest Cover | 3.3 | 0.78 | < 0.001 |
|  |  |  | (Intercept) | -1.15 | 0.62 | 0.062 |
|  | Gray Mortality ~ **Habitat Split** + Fragmentation | *Habitat Split* | *-* | *0.05* | *0.67* | *0.939* |
|  |  |  | Fragmentation | 0.35 | 0.21 | 0.098 |
| Melanic |  |  | (Intercept) | -1.78 | 1.65 | 0.28 |
|  | Melanic mortality ~ **Speed** + Human Density | *Speed* | *-* | *-0.04* | *0.02* | *0.14* |
|  |  |  | Human Density | 0.22 | 0.17 | 0.2 |
|  |  |  | (Intercept) | -4.96 | 1.7 | 0.003 |
|  | Melanic mortality ~ **Traffic** + Human Density | *Traffic* | *-* | *0.18* | *0.19* | *0.36* |
|  |  |  | Human Density | 0.32 | 0.15 | 0.037 |
|  |  |  | (Intercept) | -7.45 | 1.59 | < 0.001 |
|  | Melanic Mortality ~ **Crossings** + Human Density + Forest Cover | *Crossing* | *-* | *0.98* | *0.62* | *0.12* |
|  |  |  | Human Density | 0.5 | 0.17 | 0.004 |
|  |  |  | Forest Cover | 3.42 | 1.25 | 0.006 |
|  |  |  | (Intercept) | -2.41 | 0.93 | 0.009 |
|  | Melanic Mortality ~ **Habitat Split** + Fragmentation | *Habitat Split* | - | *1.89* | *0.92* | ***0.039*** |
|  |  |  | Fragmentation | 0.13 | 0.32 | 0.68 |

**SUPPLEMENTAL FIGURE**


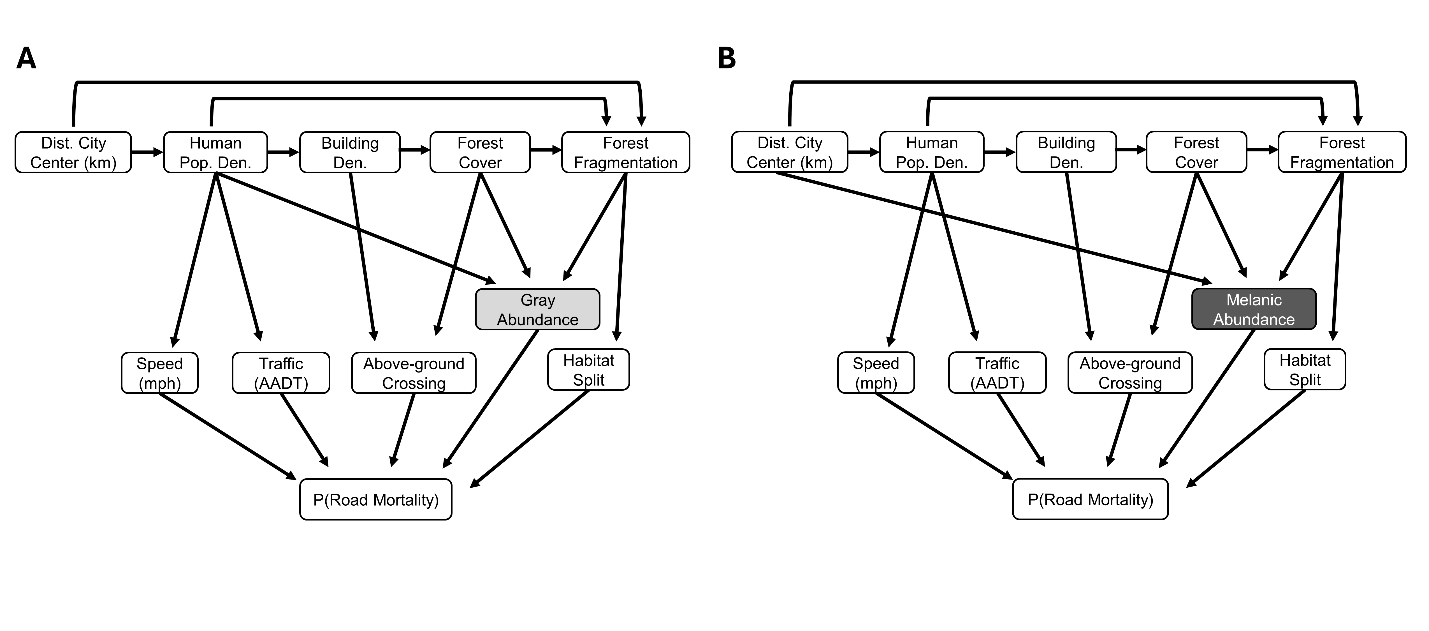


**Figure S1.** Directed acyclic graphs showing the hypothesized relationships between road and landscape covariates and probability of mortality for (a) gray and (b) melanic coat morphs and morph-specific mortality.


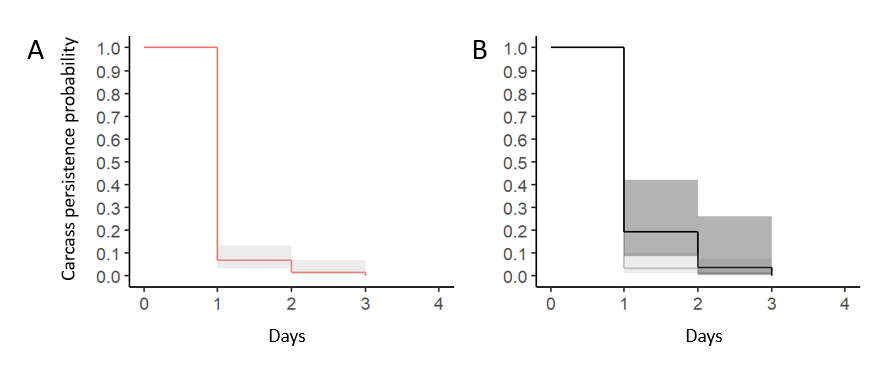


**Figure S2.** Carcass persistence of squirrel roadkill during the fall surveys. (A) Kaplan-Meier curve for squirrel carcasses combined, and (B) separate melanic and gray morph carcass persistence. A vast majority of roadkill squirrels were gone, likely scavenged, after one day with less than 10% persisting beyond a second day.
